# Three-Dimensional Blood Vessel Model with Temperature-Indicating Function for Evaluation of Thermal Damage during Surgery

**DOI:** 10.3390/s18020345

**Published:** 2018-01-25

**Authors:** Takeshi Hayakawa, Hisataka Maruyama, Takafumi Watanabe, Fumihito Arai

**Affiliations:** Room 310, Aerospace Mechanical Engineering Research Building 3F, Department of Micro-Nano Mechanical Science & Engineering, Nagoya University, Furo-cho, Chikusa-ku, Aichi-Pref., Nagoya-shi 464-8603, Japan; hisataka@mech.nagoya-u.ac.jp (H.M.); watanabe@biorobotics.mech.nagoya-u.ac.jp (T.W.); arai@mech.nagoya-u.ac.jp (F.A.)

**Keywords:** temperature measurement, surgical simulator, 3D fabrication

## Abstract

Surgical simulators have recently attracted attention because they enable the evaluation of the surgical skills of medical doctors and the performance of medical devices. However, thermal damage to the human body during surgery is difficult to evaluate using conventional surgical simulators. In this study, we propose a functional surgical model with a temperature-indicating function for the evaluation of thermal damage during surgery. The simulator is made of a composite material of polydimethylsiloxane and a thermochromic dye, which produces an irreversible color change as the temperature increases. Using this material, we fabricated a three-dimensional blood vessel model using the lost-wax process. We succeeded in fabricating a renal vessel model for simulation of catheter ablation. Increases in the temperature of the materials can be measured by image analysis of their color change. The maximum measurement error of the temperature was approximately −1.6 °C/+2.4 °C within the range of 60 °C to 100 °C.

## 1. Introduction

Recently developed medical technologies including operative procedures and medical devices are quite rapidly evolving and becoming increasingly diverse. Thus, medical doctors are required to learn these technologies with only short-term training. Additionally, medical device companies are required to develop new products within a short span of time. However, it is difficult to use the actual human body for training in new operative procedures or evaluations of new medical devices because of ethical and safety problems. Thus, there are few chances for these evaluations and it is prevented to train young doctors and accelerate the developments of new medical devices. Instead of evaluations using the human body, they are conventionally conducted with animal samples [1,2,3,4].

However, the structure of animal samples is fundamentally different from that of the human body. Furthermore, differences among individual animal samples are significant. Therefore, it is difficult to ensure reproducibility of the results of training or evaluation of medical devices using animal samples.

One solution to this problem is the use of a surgical simulator [5,6,7,8]. Two basic types of surgical simulators are available. One is a virtual reality simulator [9,10,11,12], and the other is a mock-up simulator [7,8,9]. Virtual reality simulators reproduce a sight or an environment during surgery using computer graphics. Additionally, some virtual reality simulators can reproduce the sense of touch during surgery by using haptic interfaces [12]. However, it is difficult to evaluate new medical devices using virtual reality simulators, because the effects of new medical devices on the human body cannot be reproduced completely without the accumulation of large amounts of data. Considering these limitations, mock-up simulators have been attracting increasingly more attention because they use artificial materials and can reproduce structures of the human body with high reproducibility and repeatability [13,14,15,16]. Furthermore, mock-up simulators can be used to evaluate medical devices if their characteristics are similar to those of the human body.

Although current mock-up simulators are very effective for medical doctors undergoing basic training in surgical skills such as catheter insertion, it is difficult to evaluate the thermal or electrical effects of energy devices on the human body during surgery. For example, catheter ablation, which utilizes radiofrequency electric currents, is performed to treat high blood pressure or abnormal cardiac rhythm [17,18,19]. During catheter ablation for treatment of high blood pressure or abnormal cardiac rhythm, the surgeon ablates a renal sympathetic nerve around the renal vessels or autonomous nerves of the heart, respectively. By applying a radiofrequency current with ablation catheters, the surgeon heats the diseased tissues to approximately 80 °C. This operation has a risk of thermal damage to the normal tissues around the diseased sites, and it requires a high level of surgical skill. As we mentioned before, it is difficult to meticulously evaluate this thermal damage to cells, blood vessels, or other tissues by using the actual human body. Therefore, the development of a mock-up simulator that enables evaluation of the electrical or thermal effects of such energy devices is in high demand.

**Figure 1:**
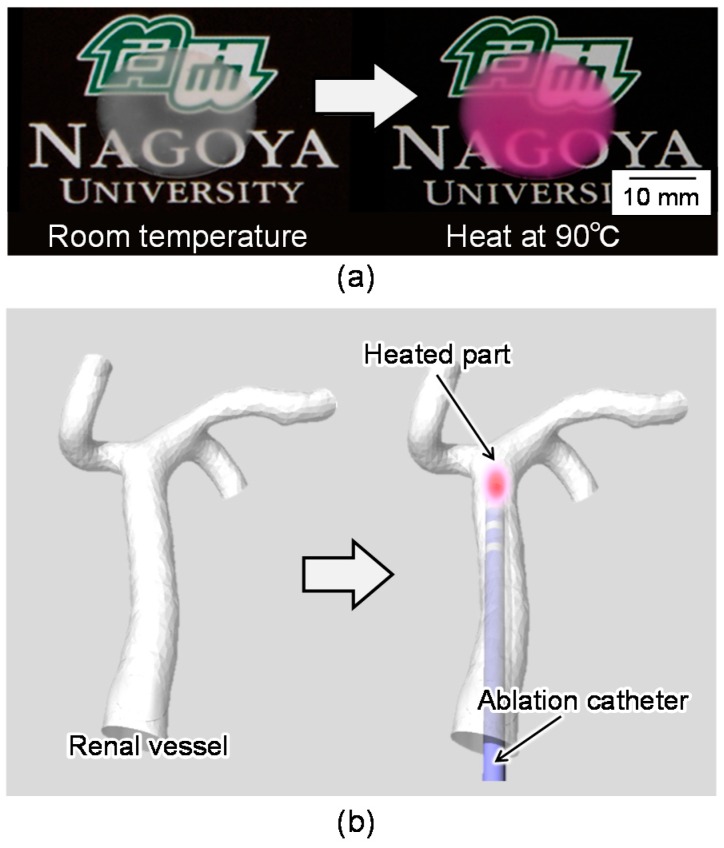
Concept of blood vessel model with temperature-indicating function. (**a**) Demonstration of color change of the composite material; (**b**) Concept of blood vessel model with temperature-indicating function.

In this study, we propose a functional mock-up simulator for the evaluation of thermal damage during surgery. We fabricated a three-dimensional (3D) blood vessel model with a temperature-indicating function to allow for evaluation of thermal damage during catheter ablation. Our model can indicate temperature increases by color changes, as shown in Figure 1. This feature is very user-friendly for medical doctors who are evaluating their surgical skills because of the visual indication of temperature increases. Furthermore, the temperature changes can be quantitatively evaluated by image analysis of color information of heated models. This quantitative indication of temperature increases is useful for evaluating the performance of medical devices such as ablation catheters.

In this report, we introduce the details of our concept in Section 2, quantitatively evaluate the temperature-indicating function of the material in Section 3, explain the fabrication process of the proposed 3D vessel model in Section 4, discuss the technical details of our proposed model in Section 5, and conclude our study in Section 6.

## 2. Concept of Functional Blood Vessel Model

We utilized a thermochromic dye to indicate temperature changes and thus evaluate the thermal damage associated with catheter ablation. Thermochromic dyes change their color according to the temperature. Thus, we established a temperature-indicating function for our surgical simulator by mixing a thermochromic dye with a base resin. This composite material (base resin and thermochromic dye) changes its color according to increases in temperature, as shown in Figure 1a. Using this composite material, we fabricated a 3D blood vessel model with a temperature-indicating function for evaluation of catheter ablation, as shown in Figure 1b.

We used polydimethylsiloxane (PDMS) (SILPOT 184; Dow Corning Toray, Tokyo, Japan) as the base resin of the model and a color-irreversible thermochromic dye (75C Magenta Irreversible WB Screen Ink; Tosco Co. Ltd., Tokyo, Japan). PDMS is a polymeric silicone used in models of the human body, because its mechanical and thermal characteristics are similar to those of the human body (Young’s modulus, 6.7 MPa; thermal conductivity, 0.27 W/m∙K) [20]. For example, the literature value of the thermal conductivity of the human epidermis is 0.21 W/m∙K, while the dermis is 0.29–0.32 W/m∙K, muscle is 0.20–0.43, and fat is 0.20–0.22 W/m∙K [21]. The color of the thermochromic dye changes from white to magenta at around 75 °C, which is near the target temperature of catheter ablation (80 °C). The color of the thermochromic dye does not revert to its original color once it changes. Therefore, the composite material has a feature of irreversible color change. Furthermore, we chose a thermochromic dye that is compatible with silicone resin so that the dye could be mixed with PDMS.

Figure 1a shows the color change of the composite material coated on a glass substrate heated to 90 °C. It takes a few tens of seconds to show this color change after application of heat. Furthermore, this color-irreversible characteristic allows our proposed model to maintain its color change at the highest temperature attained during the simulation of surgery. This feature enables post-simulation evaluation of the thermal damage that occurred during surgery. Additionally, the highest temperature can be quantitatively evaluated after the simulation by image analysis of the color of the model. This means that the proposed model can be used not only for training of doctors’ surgical skills but also for quantitative evaluation of the performance of medical devices.

## 3. Evaluation of Temperature-Indicating Function

### 3.1. Preparation of Composite Material

First, we evaluated the temperature-indicating function of the composite material. The color of the material changes from white to magenta according to the temperature increase. Therefore, the highest temperature can be measured by analyzing the color of the material after heating. In this study, we mixed the thermochromic dye with PDMS at a 2 wt % ratio. To evaluate the temperature-indicating function, we made 1.6-mm-thick sheet samples by molding. The samples were cured at room temperature for 10 h.

We also measured thermal conductivities of the original PDMS and the composite material by using thermal conductivity analyzer (TCi thermal conductivity analyzer, C-Therm Technologies, Fredericton, NB, Canada). The measured values of PDMS and the composite material were 0.161 W/m∙K (standard deviation: 0.001) and 0.151 W/m∙K (standard deviation: 0.002), respectively.

### 3.2. Measurement System Setup

To evaluate the relationship between the color of the sheet samples of the composite material and the temperature, we heated the fabricated sheet samples in 5 °C increments from 40 °C to 100 °C in a constant-temperature oven (DKM300; Yamato Scientific, Tokyo, Japan). After heating, images of the samples were acquired with a microscope (MVX10; Olympus, Tokyo, Japan) and a charge-coupled device camera (WAT-250D2; Watec Co. Ltd., Tsuruoka, Japan). The magnification of the lens was set to ×0.64, and the field of view of this setup was 7 mm high and 9 mm wide, which corresponds to 480 pixels high and 720 pixels wide. A halogen lamp (LPJP-2530 II; Hayashi Watch-Works, Tokyo, Japan) was used as a light source, and the intensity of the illumination was set to 0.03 mW/m^2^ irradiance as measured by an illuminometer (Orion PD, center of sensitive wavelength: 532 nm, Ophir Japan Ltd., Omiya, Japan).

### 3.3. Calibration of Temperature and Color Information

**Figure 2:**
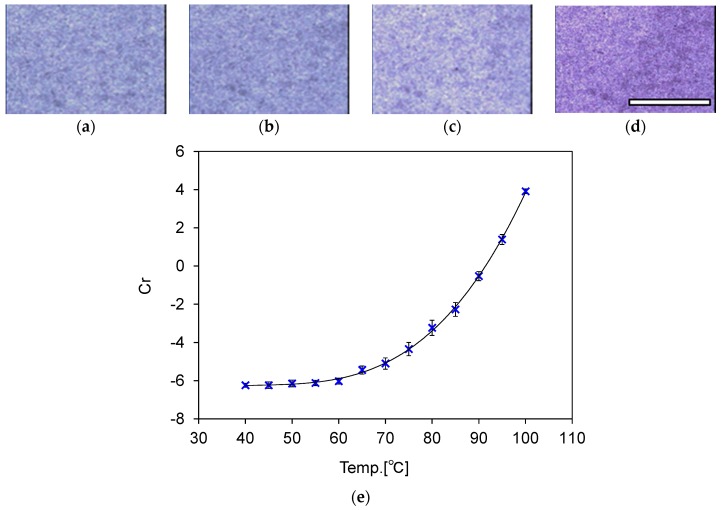
Color change of the composite material. Microscopic images of sheet samples of the composite material after heating at various temperatures. (**a**) 40 °C, (**b**) 60 °C, (**c**) 80 °C, and (**d**) 100 °C. Scale bar = 5 mm. (**e**) Calibration of temperature with *Cr* values. The solid line indicates the fitting curve of Equation (2).

The images of the samples obtained after heating are shown in Figure 2. Figure 2a–d are images of the sheet samples obtained after heating at 40 °C, 60 °C, 80 °C, and 100 °C, respectively. These images show that the color of the samples changes from white to magenta. For quantitative evaluation of the temperature increase, RGB values are obtained from these images. The RGB values are converted to *YCrCb* values with the Equation (1).

(1) Y=0.299R+0.587G+0.114BCr=0.5000R−0.419G−0.081BCb=−0.169R−0.331G+0.500B

Here, *Y* indicates brightness, *Cr* indicates the red color difference, and *Cb* indicates the blue color difference. In this study, the relationship between color and highest temperature was calibrated with *Cr* values in the *YCrCb* space, because the *Cr* values are thought to be less affected by changes in the illumination conditions [22].

The relationship between heating temperatures and *Cr* values is shown in Figure 2e. Data in this plot are the averaged values of *Cr* in the four samples. In addition, the plot is fitted with the third-order polynomial equation, shown below as Equation (2).

(2) $$Cr=5.43\times 10^{-5}T^{3}-7.11\times 10^{-3}T^{2}+\;3.16\times 10^{-1}\;T\;-\;11.0$$

Here, T indicates the heating temperature. Therefore, the highest temperature attained during heating can be calculated with Equation (2), along with the color information of the obtained images. Furthermore, measurement errors of the calculated value from the heating temperature (calculated from the deviation of the measured values in the fitting curve) are summarized in Table A1 in Appendix A. The maximum measurement error was 2.4 °C when heated to 60 °C. Additionally, the maximum measurement error at 80 °C, which is the target temperature of catheter ablation, was −0.86 °C.

From the results shown in Figure 2 and Table A1, we confirmed that the composite material of PDMS and photochromic dye can change its color according to the temperature increase and maintain this color change even after the temperature has decreased to room temperature. Furthermore, the color change can be used for measurement of the maximum heated temperature. If different temperatures are targeted in different surgeries, the threshold temperature of the color change can be modified using a different thermochromic dye [23].

Here, it may be worth mentioning a color-reversible thermochromic dye. The color-reversible thermochromic dye changes its color according to its temperature increase, the same as color-irreversible thermochromic dye. However, its color recovers to original color when its temperature decreases to room temperature. We confirmed that we were able to make color-reversible type composite material by mixing PDMS and color-reversible thermochromic dye. By using this color-reversible composite material, we can also fabricate color-reversible blood vessel model. This type of model could be used multiple times, but it is difficult to perform post-simulation evaluation of the temperature increase because the color recovers to original color. We do not evaluate this model in detail in this manuscript because our purpose of this paper is to propose the color-irreversible blood vessel model and evaluate performances of temperature measurement for post-simulation evaluation.

## 4. 3D Blood Vessel Model with Temperature-Indicating Function

### 4.1. Fabrication Process

**Figure 3:**
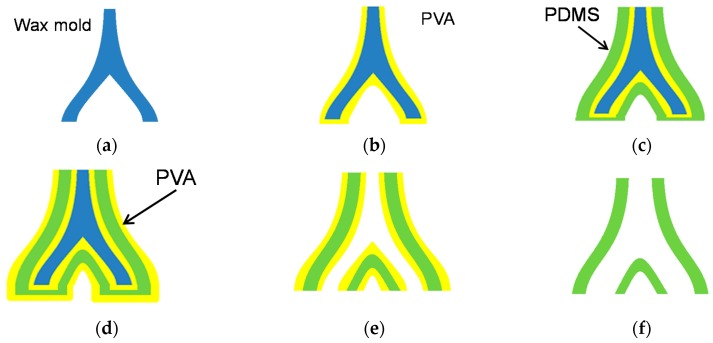
Fabrication process of 3D vessel model with temperature-indicating function. (**a**) Wax mold; (**b**) Dip-coating of PVA; (**c**) Dip-coating of PDMS with temperature-indicating function; (**d**) Dip-coating of PVA; (**e**) Removal of wax mold; (**f**) Removal of PVA.

The proposed 3D blood vessel model is fabricated with the lost-wax process, similar to a conventional blood vessel model [9]. In this study, we fabricated a renal vessel model as an example of a surgical simulator. Figure 3 shows the fabrication process of the 3D vessel model. The details are as follows:

(a) A solid wax mold is made in the shape of a blood vessel using a wax 3D printer (3Z PRO; Solidscape Inc., Merrimack, NH, USA). Structural data of blood vessel were first obtained as 2D tomographic data of DICOM format by using X-ray CT scanning. Then, 3D data of STL format were generated based on these 2D data. The data were provided by Dr. Seiichi Ikeda. For more detailed explanation about this process, please confirm Reference [14].
(b) 8 wt % polyvinyl alcohol (PVA) dissolved in water is dip-coated onto the wax mold as a protective layer. The model is then dried at room temperature for 2 h. This step is repeated four times to acquire a sufficient PVA film thickness. Evaluation of the condition of the PVA thickness is described in detail in the next subsection.
(c) The composite material of PDMS and the thermochromic dye is dip-coated onto the mold and cured at room temperature for 10 h. This step is repeated six times.
(d) 8 wt % PVA is coated onto the model four more times.
(e) The wax mold is removed using acetone.
(f) The coated PVA is removed by water.

### 4.2. Evaluation of Coating Condition of PVA

Blood vessel models can be fabricated with the lost-wax process as described above. However, organic solvent affects the temperature-indicating function of thermochromic dye. The color of the composite material changes from white to magenta when it is immersed in acetone, which is used to remove the wax mold for a short time (approximately 60 min). Moreover, the color disappears when it is immersed in acetone for a long time (approximately 1 day). Therefore, PVA is used to coat the model and preserve its temperature-indicating function. We confirmed that the PVA was well adhered to both the wax mold and PDMS, and that it remained coated until it was dissolved in water.

**Figure 4:**
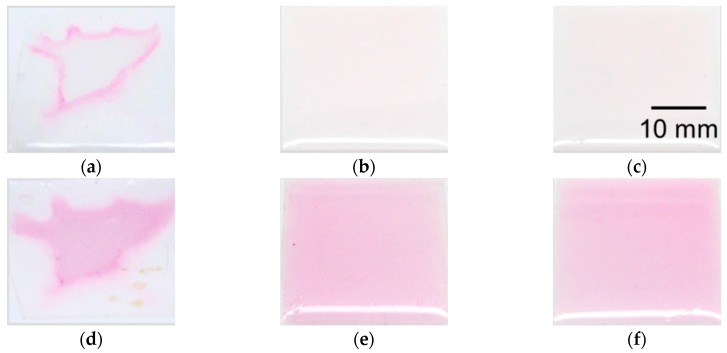
Fabricated membrane samples of the composite material with PVA coating.Samples after immersion in acetone for 1 h with PVA thickness of (**a**) 23 µm, (**b**) 47 µm, and (**c**) 85 µm. Samples after being heated at 90 °C for 1 h with PVA thickness of (**d**) 23 µm, (**e**) 47 µm, and (**f**) 85 µm.

To evaluate the coating condition of the PVA, we fabricated thin sheets of the composite material on a glass substrate. We then coated the PVA onto the fabricated membrane at various thicknesses. Next, we immersed the samples in acetone for 1 h and confirmed the color change. The evaluation results are shown in Figure 4. Figure 4a–c shows the sample with PVA thicknesses of 23, 47, and 85 µm after immersion in acetone for 1 h, respectively. PVA was coated on each sample using spin coating with rotating speeds 800, 700, and 600 rpm for 30 s. The thickness of the PVA films with each condition was measured with a microfigure measuring instrument (Surfcorder ET200; Kosaka Laboratory Ltd., Tokyo, Japan). A partial color change was observed in the sample shown in Figure 4a, which had the thinnest PVA thickness. However, no color change was observed in the samples in Figure 4b,c. Similarly, Figure 4d–f shows the sample with PVA thicknesses of 23, 47, and 85 µm after being heated at 90 °C for 1 h. As shown in Figure 4a, the color of the center part changed normally, but the color of the part around the edge did not change. Conversely, the color of the samples in Figure 4e,f changed. These results indicate that a PVA coating thicker than 47 µm is necessary to protect the temperature-indicating function of the composite material from immersion in acetone.

### 4.3. Fabrication Results

**Figure 5:**
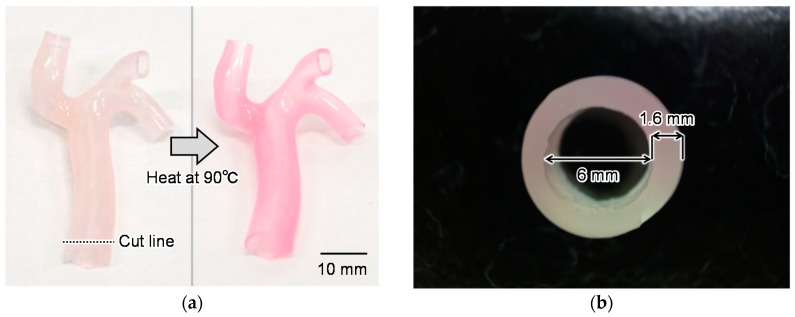
Fabricated blood vessel model with temperature-indicating function. (**a**) The vessel model before and after heating at 90 °C; (**b**) Cross-sectional view of the model cut along the cut line in (**a**).

According to the evaluation of the PVA thickness in the previous subsection, we determined the conditions of the fabrication process explained in Section 4.1. Using these conditions, we succeeded in fabricating a 3D renal vessel model with a temperature-indicating function, as shown in Figure 5a. After being heated at 90 °C, the color of the model changed from white to magenta as explained in concept. Furthermore, the thickness of the vessel model fabricated with these conditions was 1.6 ± 0.15 mm (Figure 5b), which was similar to the thickness of the sheet samples used for the temperature calibrations in Section 3. This thickness of the blood vessel model can be changed by changing the number of times the PDMS is dip-coated, as explained in the fabrication process (c). Different thicknesses of the material might have different sensitivity curves of the *Cr* values and temperature. In that case, we can measure the temperature by slicing the model with a 1.6-mm thickness.

## 5. Discussion

### 5.1. Measurement Errors and Spatial Resolution of the Temperature Measurement

Here, we discuss the relationship between the temperature measurement error by image analysis and the spatial resolution of the temperature measurement. We confirmed that the temperature measurement error at 80 °C was −0.86 °C, as shown in Table A1. This value was acquired by averaging the *Cr* values in an area of 480 × 720 pixels, corresponding to 7 × 9 mm. However, the original images had relatively large color deviations as shown in Figure 2. These color deviations are thought to be a result of dust or particles of the thermochromic dye in the model. Additionally, this deviation is thought to be the main cause of measurement errors. Thus, we acquired the *Cr* value by averaging some areas on a sample to reduce the deviation. However, the averaging of large areas produces low spatial resolution of the temperature measurement. Therefore, we evaluated the relationship between the size of the averaged area and the standard deviation of the *Cr* values, which is related to measurement error.

**Figure 6:**
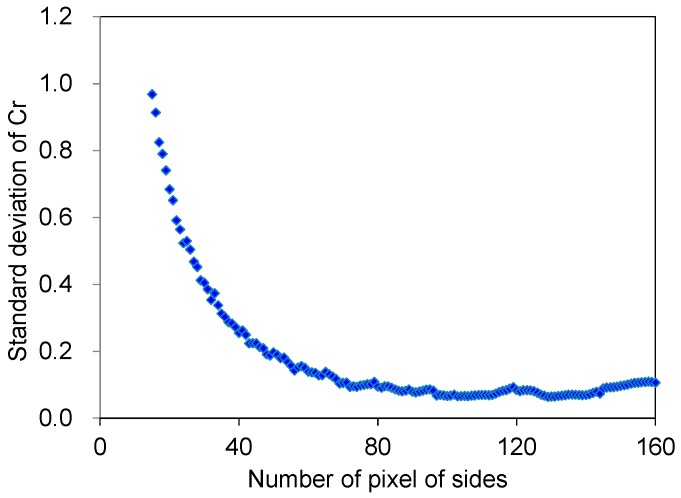
Standard deviation of *Cr* values with various averaged areas.

We fabricated four sheet samples with a 1.6-mm thickness, which is the same thickness of the samples for the temperature calibration shown in Figure 2e. The samples were heated in a constant-temperature oven set at 80 °C. After being heated for more than 1 h, images of the samples were acquired using a microscope as explained in Section 3.2. The size of the averaged area of the images varied from 1 × 1 to 160 × 160 pixels. The standard deviations of the *Cr* values were calculated and plotted in Figure 6. As shown in Figure 6, the standard deviations of the *Cr* values decrease by averaging a large area. Therefore, the measurement errors also decrease by averaging a large area. However, the spatial resolution of the temperature measurement decreased when the *Cr* values were averaged in a large area. These results indicate the presence of a trade-off relationship between the measurement error and spatial resolution of the temperature measurement. An averaged area should be chosen to satisfy the required measurement error for a specific application.

### 5.2. Evaluation of Local Heating

**Figure 7:**
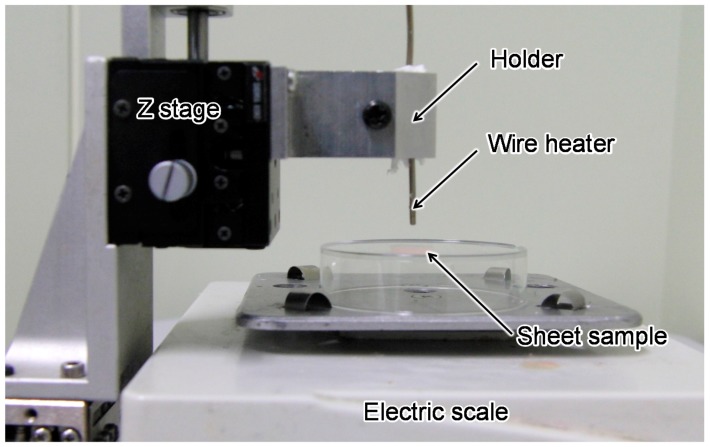
Experimental setup of local heating of sheet sample.

Finally, we demonstrated the temperature measurement of a locally heated model. We used a sheet sample of the proposed model for this evaluation to well control a heating condition. Currently, it is difficult to heat 3D blood vessel model with high repeatability. Thus, we used a sheet sample for proof of concept of proposed functional model and evaluation of basic performance of temperature measurement. The sheet sample was heated with a wire heater (heater: MBHS2.4, diameter: 2.4 mm, controller: MTCS, temperature accuracy: ±2 °C; Misumi Group Inc., Tokyo, Japan). The temperature of the wire heater was set at 80 °C, which is the target temperature of catheter ablation. The heater was fixed to a z stage and contacted the sheet sample with a force of 0.1 N for 30 s. The contact force was measured using an electric scale. The experimental setup is shown in Figure 7.

**Figure 8:**
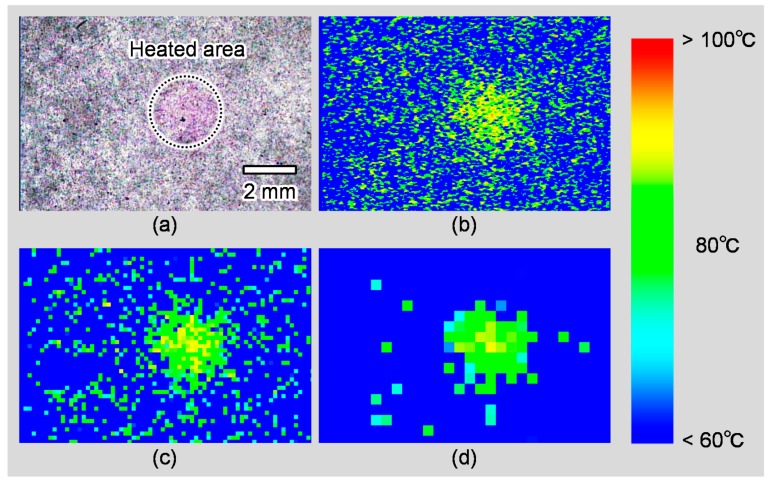
Evaluation of local heated temperature with different averaged areas. (**a**) Original picture of the sheet sample; (**b**) Analyzed image without averaging; (**c**) Analyzed image averaged with 10 × 10 pixels; (**d**) Analyzed image averaged with 25 × 25 pixels. Color bar shows the corresponding maximum heated temperature.

Figure 8 shows a picture of the heated sample and temperature distribution calculated from the picture. Figure 8b–d shows the temperature distribution without averaging, with an average 10 × 10 pixels area and 25 × 25 pixels area, respectively. From Figure 8, we confirmed that the measurement error in the non-heated area can be reduced by averaging. Furthermore, the heated area can be seen in the analyzed images of the temperature distribution shown in Figure 8b–d, and it indicates that the temperature is around 80 °C. Therefore, the proposed model with the temperature-indicating function can be used to evaluate the temperature distribution after local heating, such as catheter ablation. To construct more realistic evaluation system, which can heat 3D blood vessel model with high repeatability, is future work.

### 5.3. Thermal or Electrical Characteristics of the Model

In this manuscript, we used PDMS as base material of the proposed model, because the thermal property is similar to human body, as explained in Section 2. However, the thermal conductivities of human body are widely varied depending on tissue. And also, individual differences of thermal characteristics are thought to be large. Thus, measurement of thermal characteristics of human blood vessels and adjusting the thermal conductivity of the model to that of actual human blood vessels is needed for more realistic simulations.

Furthermore, we heated the model by using oven or wire heater to evaluate performance of temperature measurement function. In actual catheter ablation, however, the disease parts are heated by applying radiofrequency electric current. Thus, detailed heating conditions are thought to be different from our evaluation. For more realistic simulations, adjusting electrical impedance of the model to actual human blood vessels is necessary. Furthermore, it is also necessary to reproduce electrical impedance of environments.

Such detailed reproduction of thermal and electrical characteristics of the model and environments is necessary for more realistic simulations, and is the future work of this study.

## 6. Conclusions

In this study, we proposed a functional 3D blood vessel model with a temperature-indicating function that enables evaluation of thermal damage during surgery. The temperature-indicating function is realized by mixing thermochromic dye with PDMS, which is the base resin of the model. Additionally, the maximum temperature can be measured after surgical simulation, because the color of the mixed thermochromic dye is irreversible. This feature enables not only surgical training of doctors but also quantitative evaluation of the performance of surgical devices. We confirmed that the highest temperature during simulation can be measured after the simulation using color image analysis of the model. According to the calibration results, the temperature can be measured with an error within +2.4 °C/−1.6 °C. Finally, we succeeded in fabrication of the proposed 3D blood vessel model with the lost-wax process. The fabricated model indicated color changes by heating. This sensing functionalization of surgical simulators is quite important for quantitative and reproducible evaluations of surgical skills or medical device performance. Other sensing functions such as measurement of electrical or mechanical effects are also very important. Thus, these sensing functions will be reported in future studies. The proposed surgical simulator with a temperature-sensing function will strongly contribute to medicine and the medical industry, and help to realize high-quality and safe medical technologies.

## Abbreviations

The following abbreviations are used in this manuscript:

PDMS	polydimethylsiloxane
PVA	polyvinyl alcohol
3D	three-dimensional

## Appendix A

Here, we show the temperature measurement error with visual analysis at each temperature in Table A1.

**Table A1:** Temperature measurement error of calculated value and heated temperature.

Temperature (°C)	Error (°C)
60	2.40
65	−1.64
70	0.07
75	−0.26
80	−0.86
85	0.37
90	−0.11
95	0.04
100	−0.22
